# The influence of standardized dry ivy leaf extract on the proportion of nasal secretion after post-septoplasty nasal packing removal^☆^^☆☆^

**DOI:** 10.1016/j.bjorl.2018.05.005

**Published:** 2018-06-21

**Authors:** Slobodan Savović, Milica Paut Kusturica, Vladimir Kljajić, Maja Buljčik Čupić, Ljiljana Jovančević, Vedrana Pavlović, Aleksandar Rašković

**Affiliations:** a University of Novi Sad, Faculty of Medicine, Clinical Centre of Vojvodina, Ear, Nose and Throat Clinic, Novi Sad, Serbia; b University of Novi Sad, Faculty of Medicine Novi Sad, Department of Pharmacology Toxicology and Clinical Pharmacology, Novi Sad, Serbia; c University of Belgrade, Faculty of Medicine, Belgrade, Serbia

**Keywords:** Dry ivy leaf extract, Nasal secretion, Post-septoplasty nasal packing removal, Extrato de folha seca de hera, Secreção nasal, Remoção de tampão nasal pós-septoplastia

## Abstract

**Introduction:**

After post-septoplasty nasal packing removal, a certain proportion of nasal secretion occurs, leading to local and sometimes systemic infections.

**Objective:**

The aim was to determine if standardized dry ivy leaf extract application after nasal packing removal influences the reduction of nasal secretion and diminish the occurrence of local infections.

**Methods:**

The study included 70 post-septoplasty patients (divided into two equal groups) whose nasal packing was removed on the third day after the procedure. Group I was treated with standardized dry ivy leaf extract syrup along with regular nasal irrigation for the five days after the nasal packing removal whereas the Group II had only nasal lavage. On the sixth day after nasal packing removal, the quantity of nasal secretion was determined using a visual analog scale and nasal endoscopic examination.

**Results:**

The group treated with standardized dry ivy leaf extract syrup had significantly lesser nasal secretion both by subjective patients’ assessment (*p* < 0.001) and by nasal endoscopic examination (*p* = 0.003). The post-surgical follow up examination on the sixth day after nasal packing removal showed no development of local infection in the Group I, while in the Group II a local infection was evident in five patients (14.29%) and antibiotic therapy was required.

**Conclusion:**

The use of the standardized dry ivy leaf extract after nasal packing removal significantly lowers the proportion of nasal secretion.

## Resumo

**Introdução:**

Após a remoção do tampão nasal pós-septoplastia, ocorre produção de secreção nasal, predispondo infecções locais e, por vezes, sistêmicas.

**Objetivo:**

O objetivo foi determinar se a aplicação do extrato padronizado de folhas de hera seca após a remoção do tampão nasal influencia a redução da secreção nasal e diminui a ocorrência de infecções locais.

**Método:**

O estudo incluiu 70 pacientes pós-septoplastia (divididos em dois grupos iguais) cujo tampão nasal foi retirado no terceiro dia após o procedimento. O grupo I foi tratado com xarope padronizado de extrato de folha seca de hera juntamente com irrigação nasal regular por cinco dias após a remoção do tamponamento nasal, enquanto ao grupo II foi recomendado apenas lavagem nasal. No sexto dia após a remoção do tampão nasal, a quantidade de secreção nasal foi determinada pela escala EVA (escala visual analógica) e pelo exame endoscópico nasal.

**Resultados:**

O grupo tratado com xarope de extrato seco de folhas de hera apresentou secreção nasal significativamente menor tanto pela avaliação subjetiva dos pacientes (*p* < 0,001) quanto pelo exame endoscópico nasal (*p* = 0,003). O exame de acompanhamento pós-cirúrgico no sexto dia após a remoção do tampão nasal não mostrou desenvolvimento de infecção local nos pacientes do grupo I, enquanto que no grupo II, cinco apresentaram sinais de infecção local (14,29%) com necessidade de antibioticoterapia.

**Conclusão:**

O uso do extrato padronizado de folhas secas de hera após a remoção do tampão nasal reduz significativamente a produção de secreção nasal.

## Introduction

Septoplasty is one of the most common surgeries among all ENT (ear, nose, and throat) surgeries in the US.^1^

In order to have a better control over post-surgical bleeding, to prevent the occurrence of hematoma of the nasal septum as well as post-surgical adhesion and to maintain nasal septum stabilization after septoplasty, anterior nasal packing is the most frequent practice. However, there are no standardized opinions on the need of the classic nasal packing after septoplasty, the type of material used as well as its duration.^2^ Numerous authors do not recommend nasal packing after septoplasty due to a number of discomforts during the packing itself, the fear and the pain while packing removal from the nose as well as some possible systemic complications. Also, no significant difference was found between the patients who underwent nasal packing and the patients who did not in terms of post-surgical bleeding, occurrence of nasal septum hematomas and post-surgical adhesions.^3^, ^4^, ^5^ Bernardo et al. concluded that routine anterior nasal packing was not beneficial and that it can increase morbidity and potential complications.^6^

After nasal packing removal, the patients usually complain about numerous problems, most commonly the presence of excess nasal secretion. This secretion is usually the consequence of the nasal packing which has been in direct contact with nasal mucosa, leading to an augmented secretion production and the loss of cilia.^7^ Decreased number and a weakened function of cilia are followed by a decrease of mucociliar clearance that hardens secretion elimination. When there is secretion in the nasal cavity, local infection may trigger a nasal septum abscess, which can result in systemic infection complications and life-threatening conditions such as: cavernous sinus thrombosis, meningitis, brain abscess and other.^8^ Numerous studies have demonstrated that infections are rare after an elective nose surgery in otherwise healthy patients.^9^ For example, Caniello et al. have concluded that septoplasty is the procedure that does not require prophylactic use of antibiotics due to a very low post-surgical risk of an infection.^10^ On the other hand, Rechtweg et al. have found that 66% out of the queried otorhinolaryngologists routinely used antibiotics after a septoplasty in order to prevent a post-surgical infection or to avoid a toxic shock syndrome.^11^ However, the use of antibiotics can lead to unwanted antibiotic resistance, toxic reactions and enormous costs. Furthermore, the incidence of an allergic reaction is present in 0.7% to 10% of the cases, and death can occur in one out of twenty-five thousand patients.^12^

Standardized dry ivy leaf extract is a safe secretolytic that has been in long-term use to decrease secretion viscosity and secretion elimination from respiratory airways.^13^ Also, the dry ivy leaf extract is well-known for its antiinflammatory effect.^14^

The aim of this study was to explore whether the use of secretolytics after post-septoplasty nasal packing removal influences the proportion of nasal secretion, as well as whether the use of secretolytics has an impact on local infection occurrence of the nose and the paranasal sinuses in the post-surgical period in which the use of antibiotics was indicated.

## Methods

The study was performed at the ENT Clinic, Clinical Centre of Vojvodina and approved by Ethical committee of Faculty of Medicine, Novi Sad (11.05–2006). It was an open-label, prospective and randomized study. The research was performed according to the Helsinki declaration principles. The patients were informed about the research and signed the informed consent after having understood the procedure. All the patients were free to quit the participation in the study at any moment.

Sample size calculation was based on requirement to reveal differences in the subjective and endoscopic assessment of the quantity of nasal secretion between two treatment options, using the Mann–Whitney test. The sufficient total sample sizes providing at least 0.8 powers, at 0.05 significance level, to reveal an average differences in subjective assessment score of 2 and endoscopic score of 1, were 38 and 42 subjects, respectively. Sample size calculation was conducted using the R package “WMWssp”.^15^ Patients involved in the research (47 men and 23 women) ranged in age from 18 to 56. All the patients included in the study had undergone a septoplasty due to nasal septum deformity and were diagnosed on the basis of their case histories, anterior rhinoscopy, nasal endoscopy, anterior rhinomanometry and acoustic rhinometry. The patients with allergic rhinitis, chronic rhinosinusitis, nasal polyposis, known immunodeficient status as well as those who had undergone any kind of nose or paranasal surgeries in the past were not included in the study. Also, the patients who underwent inferior turbinoplasty were not included in the study.

The surgeries were performed under general anesthesia with orotracheal intubation and had anterior nasal packing inserted. Septal cartilage in the collumelar pocket was fixated by Vicryl (3–0) sutures in all the patients, while hemitransfixion was fixated by silk (2–0) sutures. Silk sutures were removed on the seventh day after septoplasty. The nasal packing was done with vaseline gauze dressing. Nasal splints were not used in any patients. All the patients had the nasal packing removal on the third postsurgical day. Antibiotics were not administered to any of them. For the purpose of the study, dry ivy leaf extract syrup [Prospan® Syrup (containing 7 mg of dry ivy leaf extract/mL)] was taken orally.

The patients were randomly divided into two equal groups of 35 patients. The first group (Group I) consisted of 35 patients (23 men and 12 women) who used saline nasal lavage (5 times a day 10 mL in each nostril) along with taking standardized dry ivy leaf extract syrup, for five days after nasal packing removal. They were treated with 105 mg of dry ivy leaf extract (15 mL of syrups a day) divided into three doses of 5 mL (one teaspoon i.e. 35 mg of dry ivy leaf extract). The other group (Group II) consisted of 24 men and 11 women. They had only nose lavage for five days after nasal packing removal. On the sixth day, the patients estimated the quantity of nasal secretion after nasal packing removal on the 0–10 scale, 0 being secretion free and 10 being totally secretion obstructed. Also, on the sixth post-nasal packing removal day, nasal endoscopic examination was done to determine the degree of secretion.

The secretion proportion was assessed on the 0–4 scale in which 0 indicated the absence of secretion, 1 small amount, 2 moderate, 3 moderate to large amount, 4 massive.^10^

Statistical analysis was performed with IBM SPSS Statistics 21. Results were presented as arithmetic mean, median and measures of variability (standard deviation and range). Student's *t*-test and non-parametric Mann–Whitney test were used to test the differences in numerical features between two independent samples. The chi square test was used to test the differences between nominal data. Spearman correlation coefficient was computed to assess the relationship between two variables. All *p*-values <0.05 were considered significant.

## Results

Group I (patients who received standardized dry ivy leaf extract syrup after nasal packing removal and nasal lavage) consisted of 23 men and 12 women of the average age of 32.80, while Group II (only the nasal lavage) consisted of 24 men and 11 women of the average age of 30.06. There were no significantly statistical differences in gender (*p* = 0.799) and age (*p* = 0.960) between the two examined groups.

Significantly higher values in nasal secretion subjective assessment were registered on the sixth post-surgical day in Group II (received only medical nose lavage) in comparison with group I (received standardized dry ivy leaf extract syrup) (*Z* = 4.188, *p* < 0.001) (Table 1). Also, in Group II statistically significant greater secretion values were found endoscopically on the sixth day after nasal packing removal (*Z* = 3.014, *p* = 0.003) in relation to the patients who were given standardized dry ivy leaf extract along with the nasal lavage – Group I (Table 2).

**Table 1:** Subjective assessment of the quantity of nasal secretion.

Group	$\bar{x}$	Median	SD	Min	Max
I	1.34	1.00	1.23	0	5
II	3.29	3.00	2.07	0	8
Total	2.31	2.00	1.95	0	8

Group I, standardized dry ivy leaf extract and nasal lavage group; Group II, only nasal lavage group.

**Table 2:** Endoscopic finding of the quantity of nasal secretion.

Group	$\bar{x}$	Median	SD	Min	Max
I	0.69	1.00	0.76	0	2
II	1.46	1.00	1.12	0	4
Total	1.07	1.00	1.03	0	4

Group I, standardized dry ivy leaf extract and nasal lavage group; Group II, only nasal lavage group.

Positive correlation was found between the subjective assessment and nasal endoscopic findings of nasal secretion in both groups of patients: Group I (*ρ* = 0.956, *p* < 0.001), Group II (*ρ* = 0.731, *p* < 0.001).

The post-surgical follow up examination on the sixth day after nasal packing removal showed no development of local infection in Group I and no need for antibiotic therapy, while in Group II a local infection developed in five patients (14.29%) and antibiotic therapy was required. This difference was statistically significant (*x*^2^ = 5.385, *p* = 0.020). Systemic infection was not developed in any of the examined patients.

## Discussion

Like any other surgical procedure, septoplasty can be followed by possible complications, such as local infection that can occur in the surgical region or very rarely systemic infections as meningitis, cavernous sinus trombosis, brain abscess, which can be life-threatening.^16^ Based on the literature data Georgiou et al. concluded that infections occurred very rarely after elective surgeries in rhinology.^9^ During or after the septoplasty, most authors did not find a significant difference in infection frequency between the patients treated with antibiotics and those not treated with antbibiotics.^10^, ^17^, ^18^ Ricci and Ascanio and Caniello et al. have not revealed any statistically significant difference in the proportion of purulent secretion, which is considered a sign of the local infection, between the patients who were post-surgically administered antibiotics and the ones who were not.^10^, ^19^ Moderate to large or massive secretion quantity (stages 3 and 4) was not observed in any of the patients in both groups. However, in the study conducted by Lilja et al. it was noticed that the infection was more frequent in the patients who were not treated with antibiotics. In the group of surgical patients, 3 nasal septum abscesses were found, and none of them were treated with antibiotics.^18^ However, the difference between the groups was not statistically significant, which can be explained by a small number of examined patients. Gioacchini et al. have concluded that there was no need for routine use of antibiotics after septoplasty, except in some cases such as cardiosurgical interventions, immunosuppresive conditions and similar procedures.^20^ In our study, antibiotics were not administered to any patient after septoplasty. The vast majority of rhinologists use classic nasal packing after septoplasty, primarily to prevent the occurrence of post-surgical bleeding, synechiae and hematoma of the nasal septum.^20^, ^21^

In our study, every patient underwent classic nasal packing which was removed on the third post-surgical day. The patients often complained about the nasal packing removal secretion. This secretion is most frequently the consequence of prior nasal packing presence that was closely connected to nasal mucosa, leading to mucosal damage, the loss of cilia and increased secretion production.^7^, ^22^ Ohashi et al. have found that recovery after nasal mucosa injury occurred after five days unless the basement membrane was injured along with the basal cells.^23^ Otherwise, the ciliary apparatus will start regeneration three weeks after the injury, and total recovery can be expected in 6 weeks. Kula et al. have not found that nasal packing leads to the ciliary apparatus of nasal mucosa damage.^21^ However, the examination was performed 6 weeks after the nose surgery when nasal mucosa was already fully recovered. The decrease of cilia number and their weakened function resulted in decrease of mucociliary clearance, which hardened the increased secretion quantity elimination. When this secretion is eliminated from nasal cavity, as is mentioned before, local infections, nasal septum abscesses and some life-threatening conditions may develop.

In the present study, the patients who were administered standardized dry ivy leaf extract syrup along with nasal lavage reported a statistically significant lesser quantity of nasal secretion in comparison to those who had undergone nasal lavage only. Also, the nasal endoscopic examination on the sixth day after nasal packing removal showed statistically significant lesser quantity of nasal secretion in the patients who were administered standardized dry ivy leaf extract syrup along with nasal lavage. In addition, we found a statistically positive correlation between subjective nasal secretion assessment by the patients themselves and nasal endoscopic findings in both groups of patients. In the group that underwent nasal lavage only, five patients required antibiotic therapy on the sixth day after the nasal packing removal. In the group that received standardized dry ivy leaf extract, antibiotic therapy was not required. This difference was statistically significant. The guideline for antibiotic therapy introduction was the occurrence of purulent secretion in the nose along with elevated body temperature (over 38 °C) and the feeling of pain or facial pressure, especially on one side of the face.^24^ Similar results were obtained by Federspil et al. who treated acute non-purulent sinusitis with secretolytic Myrtol and vasoconstrictor xylometazoline for 6 days approximately in one group, while the other group received placebo along with vasoconstrictor.^25^ In the Myrtol group 7.3% of patients required antibiotic therapy, while in the placebo group antibiotic therapy was necessary in 12.6% patients. Tarantino et al. reported the advantage of secretolytic administration in comparison to placebo in eliminating nasal secretion in patients with rhinosinusitis, while Szmeja et al. have confirmed that secretolytic administration along with standard therapy shortens the duration of recovery in rhinosinusitis patients.^26^, ^27^ On the other hand, Van Bever et al. have not confirmed the advantage of secretolytic administration compared to saline administration in eliminating nasal secretion in juvenile patients with rhinosinusitis.^28^

Considering that the surgical procedure causes greater or lesser damage of mucociliary clearance, and consequently greater or lesser nasal discharge, we are of the opinion that the administration of Prospan® would be useful due to its secretolytic and anti-inflammatory effects, even in patients who did not undergo nasal packing after septoplasty.

In our study, none of the patients who were administered standardized dry ivy leaf extract syrup have reported any gastrointestinal or other side effects. These results are in confirmation of the studies of other authors and confirm a good tolerance and no digestion problems with herbal secretolytics.^13^, ^25^

## Conclusions

To conclude, in the present research, standardized dry ivy leaf extract administration after removing post-septoplasty nasal packing, along with standard nasal lavage, significantly decreases the proportion of nasal secretion and thus diminishes the possibility of local infection occurrence and the need for antibiotic therapy. However, despite the results of our study, major studies need to be conducted in order to confirm our findings.

## Funding

This work was supported by the 10.13039/100007225Ministry of Science and Technological Development, Republic of Serbia, Project no. 41012.

## Conflicts of interest

The authors declare no conflicts of interest.
